# Visuospatial information transfer and task self-assessment within and between autistic and non-autistic adults

**DOI:** 10.1371/journal.pone.0329825

**Published:** 2025-08-14

**Authors:** Charlotte E. H. Wilks, Sarah J. Foster, Michelle Dodd, Sue Fletcher-Watson, Martin Lages, Danielle Ropar, Noah J. Sasson, Catherine J. Crompton

**Affiliations:** 1 Centre for Clinical Brain Sciences, University of Edinburgh, Edinburgh, United Kingdom; 2 School of Behavioral and Brain Sciences, The University of Texas at Dallas, Richardson, Texas, United States of America; 3 Salvesen Mindroom Research Centre, Centre for Clinical Brain Sciences, University of Edinburgh, Edinburgh, United Kingdom; 4 School of Psychology and Neuroscience, University of Glasgow, Glasgow, United Kingdom; 5 School of Psychology, University of Nottingham, Nottingham, United Kingdom; Father Muller Charitable Institutions, INDIA

## Abstract

Previous research has demonstrated that autistic people transmit verbal information as effectively as non-autistic people; however, when autistic and non-autistic people interact less information is transmitted. We tested whether these findings generalised to a task requiring the transmission of primarily visual information and examined how accurately participants self-assessed their performance. 310 adults (154 autistic) were allocated to one of three, six-person diffusion chain conditions: (i) autistic, (ii) non-autistic, (iii) mixed autistic and non-autistic. Participant 1 in each chain watched a video of an experimenter creating a dog shape from a puzzle toy that could be manipulated. Participant 1 showed Participant 2 how to make a dog shape, Participant 2 showed Participant 3, and so on until the end of the chain. Objective Performance was scored as the number of puzzle pieces in the correct location; self-assessment was measured on a 100-point scale, and the similarity of this self-assessment was calculated by comparing it to Objective Performance. Analyses indicated no difference in the amount of information transmitted between autistic, non-autistic, or mixed chains, or in self-assessment ratings and the similarity of these. Both autistic and non-autistic participants shared information with others and evaluated their performance similarly, aligning with previous work on the transmission of verbal information. However, the predicted breakdown in information sharing in the mixed chains did not occur. It is possible that a mismatch in neurotype may not impact information transmission that is less-verbal and more visuospatial. The heterogeneity of the sample may also have overshadowed any effect of neurotype.

## Introduction

The deficit-model of autism assumes that difficulties in communication between autistic and non-autistic people arise due to autistic social impairments [1]. However, recent research examining same- and mixed-neurotype interactions challenges this assumption, suggesting that cross-neurotype social difficulties result from a mismatch between autistic and non-autistic communication styles. For example, autistic communication may be more direct and literal – a style potentially deemed rude by non-autistic people [2,3]. This may lead to autistic people feeling more comfortable and relaxed around other autistic people [3–7] and thus preferring to spend time with them [8,9]. Conversely, when autistic people interact with non-autistic people their behaviours, social judgements, and resulting misunderstandings can exacerbate autistic social difficulties [1,8,10].

Empirical evidence of communicative success between autistic partners, and selective breakdowns in communication between autistic and non-autistic partners, was published by Crompton et al. [11]. The authors used a diffusion chain paradigm [12,13] to measure the transmission of verbal information within “chains” of eight people where (a) all participants were autistic (b) all participants were non-autistic, and (c) participants alternated between autistic and non-autistic. Results indicated that autistic people transferred information to and from other autistic people as effectively as non-autistic people transferred information with non-autistic. However, information sharing broke down – that is, significantly less information was transferred – in autistic and non-autistic alternating chains. In Crompton et al. [11] participants transferred a fictional story, therefore this information was specifically language-based and necessitated verbal communication and recall. In the current study, we investigate whether the results generalise to a primarily visuospatial task with a lower verbal communication requirement. This is particularly important as a dissociation between verbal and visuospatial processing in autism is well documented. Specifically, studies have shown relatively poorer performance on language than non-language components of IQ tests [14–17]. Therefore, it may be that the added difficulty of the language-based task in Crompton et al. [11], combined with interacting with a non-autistic person, resulted in the breakdowns in communication in the mixed chains. By using a task with lower demands on verbal processing, we can explore if reduced information transfer between autistic and non-autistic partners is robust or context specific.

Participants in Crompton et al. [11] were not required to tell the story verbatim and were free to individualise the language used to describe events. This verbal task facilitated unrestricted back-and-forth conversation between dyads in the diffusion chain, i.e., one participant (the speaker) was always tasked with retelling the story, but their partner (the listener) could interject. As autistic and non-autistic people may display divergent speaker and listener behaviours [18] these differences could have underpinned the reduced information transmission in the mixed group. Such behaviours may encompass “unconventional” language use in autistic people (see Luyster et al. [19]) including overly formal “pedantic” language [20], and repeating words and phrases [21]. It has also been suggested that autistic word choice irregularities may be characteristic of an autistic style of speaking, a “linguatype”, which might involve using language differently/more creatively [22,23]. Moreover, autistic people are less likely to fill natural pauses in spoken language with discourse markers, such as “um”, [24–26] which in non-autistic speech are important for signalling subtle elements of pragmatics to the listener (e.g., indicating desire to continue speaking, expressing uncertainty, drawing attention) and act to maintain discourse and aid conversational reciprocity [27]. Autistic people also provide less listener feedback than non-autistic people: they are less likely to comment on [28] or acknowledge a speaker’s utterance [29]. Contrastingly, in non-autistic speakers, listener feedback is frequently used to show attention, interest, and understanding in what is being said [18] and a lack of such feedback can cause speakers to feel less comfortable, and result in less efficient language use [18,30,31]. While autistic people may empathise more with autistic others [32], and be more likely to accommodate non-conventional conversational behaviours from autistic people [33], this may not transfer across to interactions with non-autistic people, potentially leading to cross-neurotype communication breakdowns. We examine information transfer in a situation that does not solely depend on language and in which performance is measured on a visuospatial task, therefore allowing us to analyse how a different type of information is transferred in same and mixed neurotype groups.

Other factors may affect communication and interaction between autistic and non-autistic people, including whether interlocutors are aware of the diagnostic status of their partner [34]. Awareness of autism status influences how autistic people are perceived by non-autistic, but not other autistic people [8,9], however many autistic adults choose not to disclose their diagnosis to avoid bias and discrimination [35]. To examine the role of diagnostic disclosure, we included a manipulation in which some participants were informed of the diagnostic status of their partners, while others were not, to examine whether neurotype awareness has effects on information transmission and self-assessment.

While research on how autistic people are perceived by others has grown in recent years [2,35–37], autistic self-perception remains under-examined. Self-assessment refers to an ability to accurately gauge one’s level of task performance and is considered an aspect of metacognition [38]. Under- or over-assessing one’s abilities can have a detrimental influence, not only on current task performance, but on broader functional outcomes [39]. Moreover, in non-autistic people, reduced self-assessment accuracy can predict functional outcomes better than objective performance [40,41]. The literature relating this area to autism is sparse – studies on autism and metacognition often focus on assessment of one’s own mental state [42], social competence [43], or memory [44], rather than performance. A recent study examined self-assessment and performance on specific general and social cognitive tasks, finding evidence that autistic adults were less accurate at assessing their performance on social, but not general, compared to non-autistic adults [38]. Accurate self-assessment may also be important for changing one’s behaviour – and thus outcomes – in “real time” during interactions such as interviews, meetings, or during a collaborative task. We thus examined the similarity of participants’ self-assessment by comparing it to their objective performance.

In summary, this study examines how participants in autistic, non-autistic and mixed diffusion chains perform on a visuospatial learning task, assessing whether a same-neurotype benefit as found in Crompton et al. [11] generalises to a task with lower language demands. Hypotheses include: (H1) mixed chains will show lower objective performance than autistic and non-autistic chains; (H2) autistic groups will perceive their performance as significantly lower, and non-autistic significantly higher, than mixed groups; and (H3) autistic chains will have poorer similarity ratings than the mixed and non-autistic. Additionally, we examined whether being informed of the diagnostic status of your group affected task performance/perceived performance as an exploratory and undirected hypothesis.

## Methods

### Ethics

Ethical approval was obtained from the University of Edinburgh’s Medical Research Ethics Committee (21-EMREC-036), the University of Nottingham’s School of Psychology Ethics Committee (F1381), and the University of Texas at Dallas’s Institutional Review Board (IRB-21–497). All participants provided written informed consent and were compensated for their time (£30/$40). Data collection occurred across three sites: the University of Edinburgh (1^st^ September 2022–1^st^ December 2023), the University of Nottingham (30^th^ October 2022–1^st^ November 2023), and the University of Texas at Dallas (1^st^ September 2022–1^st^ November 2023).

### Participants

Participants were recruited through mailing lists, local charities and community networks, social media, University research databases, and a dedicated project website. All were aged 18 + , native-level English speakers, and had normal/corrected to normal vision and hearing. Participants initially completed an online survey administered through Qualtrics to enable collection of demographic data and ensure eligibility; those with a diagnosis of social anxiety or uncontrolled epilepsy were not eligible.

Autistic people were either clinically diagnosed (n = 114) or self-diagnosed (n = 40). Non-autistic and clinically diagnosed autistic participants completed the Ritvo Autism and Asperger’s Diagnostic Scale 14-item Screen (RAADS-14 [45]); non-autistic people were excluded if their scores indicated high levels of autistic traits (score ≥ 14). Self-diagnosed autistic participants instead completed the more comprehensive Ritvo Autism and Asperger Diagnostic Scale (RAADS-R [46]) and were included if their score was above 72, indicating a high level of autistic traits [47]. RAADS-14 scores for self-diagnosed autistic participants were derived from their RAADS-R scores, and did not differ from that of clinically diagnosed autistic participants (self-diagnosed: mean = 31.46, SD = 7.06; clinically diagnosed: mean = 40.77, SD = 37.61; p = .748).

The final sample consisted of 310 participants (154 autistic, 156 non-autistic). We did not identify and exclude any outliers (defined in our pre-registration as values ±2.5 standard deviations from the mean for our objective and subjective performance variables). The autistic group was predominantly female (51.30%) and non-binary (30.52%) with a mean age of 28.68 years (SD = 11.18); the non-autistic group was predominantly female (75.64%) with a mean age of 26.87 (SD = 11.29). Autistic and non-autistic participants significantly differed on gender (p < .001; more autistic participants identified as non-binary), age (p = .020; autistic participants were older), IQ (p < .001; autistic participants had a higher IQ), and ethnicity (p < .001; more autistic participants were white) therefore these control variables were included in post-hoc analyses (see Supplementary S7 and S8 Tables). Full participant details are reported in Table 1.

**Table 1:** Descriptive statistics and group comparisons for demographics, IQ, and clinical information by diagnostic status. Categorical variables were compared using Fisher’s exact test, and continuous variables were compared using Wilcoxon Rank-Sum Test.

Characteristic	Autistic (*n *= 154)	Non-Autistic (*n *= 156)	p-value
**Age (years)**	28.68 ± 11.18	26.87 ± 11.29	.020
**Gender**			<.001
Woman	79 (51.30%)	118 (75.64%)	
Man	23 (14.94%)	34 (21.79%)	
Non-binary/gender neutral	47 (30.52%)	3 (1.92%)	
Prefer not to disclose	3 (1.95%)	1 (0.64%)	
Prefer to self-describe	2 (1.30%)	0	
**Ethnicity**			<.001
White	116 (75.32%)	87 (55.77%)	
Asian	14 (9.09%)	50 (32.05%)	
Black	8 (5.19%)	5 (3.21%)	
Mixed or Multiple Ethnicities	11 (7.14%)	8 (5.13%)	
Hispanic	3 (1.95%)	2 (1.28%)	
Other	2 (1.30%)	4 (2.56%)	
**IQ-WASI-II** ^**a**^	115.62 ± 13.59	109.86 ± 11.49	<.001
**Autistic Traits- RAADS-14** ^**b**^	33.39 ± 18.59	5.13 ± 4.17	<.001
**Age of Diagnosis (years)**	23.72 ± 12.68	NA	

^a^ IQ as assessed by the Wechsler Abbreviated Scale of Intelligence II (WASI-II)

^b^ Autistic traits, as assessed by the Ritvo Autism and Asperger’s Diagnostic Scale-Revised-14 item screen

### Measures and procedures

#### Chain types.

Participants were allocated to one of three conditions: autistic (*n* = 103), non-autistic (*n* = 103), and mixed (*n* = 104: 52 autistic and 52 non-autistic). This resulted in 54 diffusion chains: 18 in each condition. Most chains included six participants but 14 contained five (5 autistic, 5 non-autistic, 4 mixed) due to unforeseen participant non-attendance. Within each chain, participants were placed into ascending age order to minimise any potential confounding effects of age-related memory and executive function decline which could affect ability to recall, plan and organise information [48]. Switches by gender were minimised – i.e., most interacting participants were the same gender – to avoid any effect of mismatched gender impacting information transfer and rapport [12]. We ensured that participants did not know the person they were interacting with.

#### Disclosing diagnostic status.

206 participants (36 chains; 12 in each condition) were allocated to an informed condition, and 104 participants (18 chains; 6 in each condition) to an uninformed condition, in which they were respectively informed or not informed of the diagnostic status of others in their chain.

#### Randomisation.

Participants were assigned to an autistic, non-autistic or mixed chain type, and an informed or uninformed condition, in a non-randomised fashion according to order of recruitment and their ability to attend on a particular date (in addition to the age and gender restrictions outlined above) i.e., we ran a chain of a particular condition when we had recruited enough participants of the correct neurotype available on the same date.

#### Procedure.

**Fig 1:**
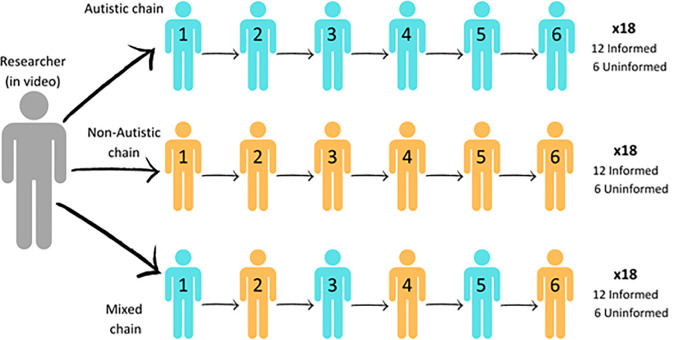
Illustration of the diffusion chain method and the number of chains of each type (Autistic, Non-Autistic, Mixed; Informed, Uninformed) included.

Data was collected across three sites: the University of Texas at Dallas (*n* = 104), the University of Edinburgh (*n* = 100), and the University of Nottingham (*n* = 106). The Rubik’s Twist Task employed a diffusion chain method in which groups of six participants transmitted their attempted solutions to a novel problem from one to another along a chain (Fig 1). This method is commonly used to investigate cultural learning in social groups and has been described as an experimental form of the game “Telephone” because the first participant transmits information to the second participant, who transmits information to the third, and so on [11].

**Fig 2:**
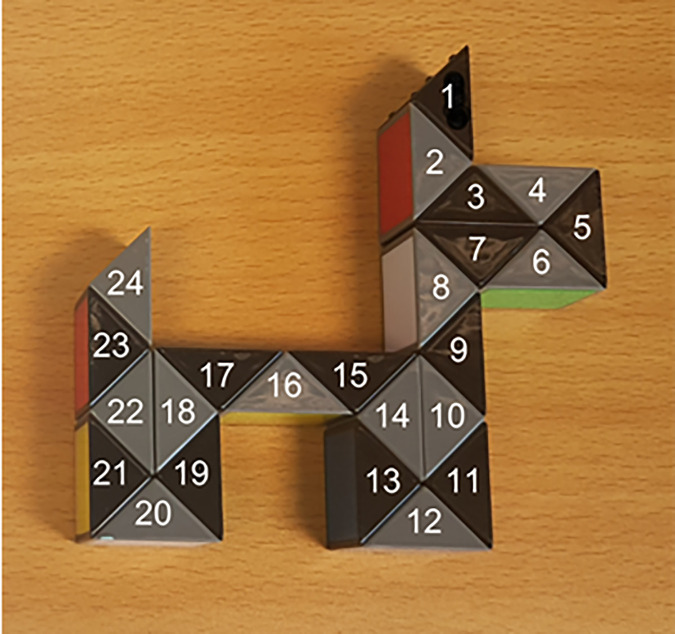
Example photograph of the dog shape created by the experimenter and shown to Participant 1 in all diffusion chains. The correct position of the 24 prism-shaped wedges is denoted by numbers 1-24; these were not visible to participants and are superimposed here to illustrate neighbouring wedges.

The first participant (Participant 1) watched a video of a female researcher creating a dog shape from a 3D puzzle toy known as a Rubik’s Twist. This consisted of 24 prism-shaped wedges that could be twisted or turned to create a wide range of shapes (Fig 2). The participant was told that they would have one minute to practise creating the dog shape, before Participant 2 entered the room. Participant 2 was subsequently told that they were going to watch Participant 1 make a dog shape and that they would then have one minute to practise before showing the third participant. The experimenter left the room whilst participants 1 and 2 interacted; after three minutes had elapsed, they returned to the testing room and photographed the final shape created. Participant 1 was then taken to an individual room to complete a self-rating task. Meanwhile, Participant 2 practised making a dog shape and was then asked to demonstrate for the third participant. This cycle of practice and demonstration continued until the sixth (final) participant, who was directed to produce a dog shape for the video camera rather than a person. All participants could indicate that they had finished early by opening the testing room door to prompt the experimenter to re-enter. Participants each waited in separate rooms for their turn to avoid contamination during the information sharing. Half of the mixed chains began with an autistic participant, and half with a non-autistic participant; the chain then alternated between autistic and non-autistic participants. The instructions given to participants can be found in the Supplement.

#### Rubik’s twist task objective performance.

Participants’ performance (henceforth “objective performance”) was scored using the photos of the dog shapes they produced but substituted stills from the videos when no photo was available, or it was of insufficient quality. Objective performance was calculated as the number of wedges in the same position as the video and scored on a scale of 0–24 then converted into a percentage. The full coding scheme, and examples of dog shapes produced by three participants, is available in the Supplement (pages 10-11) and S1 Fig. Higher objective performance scores indicated that a greater amount of information had been retained and transmitted. One researcher independently coded all 310 dog shapes produced by participants. A second researcher then re-coded 33 randomly selected dog shapes (11 from each site), giving a 10.6% overlap of videos that were double coded. Inter-rater reliability was calculated using a Single Rating Absolute-Agreement 2-Way Mixed-Effects model as per Koo and Li (2016) [49]: ICC 0.903, F = 19.3, p < .0001, 95% CI [0.814, 0.951].

#### Self-assessment.

Following completion of the Rubik’s Twist task, participants 1–5 (or 1–4 in 5-person chains) individually self-rated how much they agreed with the following two statements on a scale of 0–100: *I did what I was meant to do* (Statement 1)*; I am pleased with what I did* (Statement 2). A Pearson correlation coefficient between Statement 1 and Statement 2 indicated a strong positive correlation (r(N = 255) =.87, p < .001), therefore we used the mean of the two statements as a measure of “Subjective Performance” i.e., an individual’s view of their own task performance. In order to also capture how accurately participants’ were perceiving their individual task performance, we created a new variable named “Rating Similarity” by calculating the difference between participants’ aforementioned Objective Performance (0–100) and Subjective Performance (0–100) as follows: (Subjective Performance (0–100)) − (Objective Performance (0–100)) = (Rating Similarity (−100–100)). A Rating Similarity of zero therefore indicates that a participant self-rated with perfect similarity, whilst a positive value indicates an over-rating of one’s performance (i.e., Subjective Performance > Objective Performance) and a negative value an under-rating (i.e., Objective Performance > Subjective Performance). The final participant in each chain did not complete this measure because it was part of a questionnaire completed after the experimental task about participants’ experience producing a dog shape for the subsequent participant in the diffusion chain. As Participant 6 was the final participant in the chain, they produced a dog shape in front of a camera, rather than a participant. Therefore, they did not complete this measure. The number of participants who provided this data is therefore 255 (128 autistic, 127 non-autistic).

#### IQ.

To characterise the IQ of the sample, participants completed the Wechsler Abbreviated Scale of Intelligence II two item subtest (WASI-II [50]).

### Statistical analysis

All analyses were pre-registered on the OSF (https://osf.io/t7ng2/?view_only=b205601a48e743c99b2a4f5f5e69cd34) and conducted in R [51]. Data and the R code required for reproducing the analysis can be found here: https://osf.io/t7ng2/?view_only=b205601a48e743c99b2a4f5f5e69cd34. Linear mixed effects, and standard linear regression, models were used for analysis and are described in further detail in the below results section and in the Supplement.

## Results

### Objective performance

To examine whether Objective Performance was lower in mixed chains compared to the autistic and non-autistic chains (H1), linear mixed effects models were used (LMM R-package lme4 [52]). The best fitting model in terms of Akaike Information Criterion (AIC) and Variance Inflation Factor (VIF) for the dependent variable of Objective Performance included predictor variables of Chain Type (fixed effect; 3 levels; between: Autistic, Non-autistic, Mixed), Chain Position (fixed effect; within: 1–6), Diagnostic Informing (fixed effect; 2 levels; between: Informed, Uninformed), and a by-Chain ID random intercept and slope for Chain Position. This model predicted task performance with an adjusted (marginal) R² = 0.341 and adjusted (conditional) R² = 0.084 (R-package MuMIn [53]).

**Fig 3:**
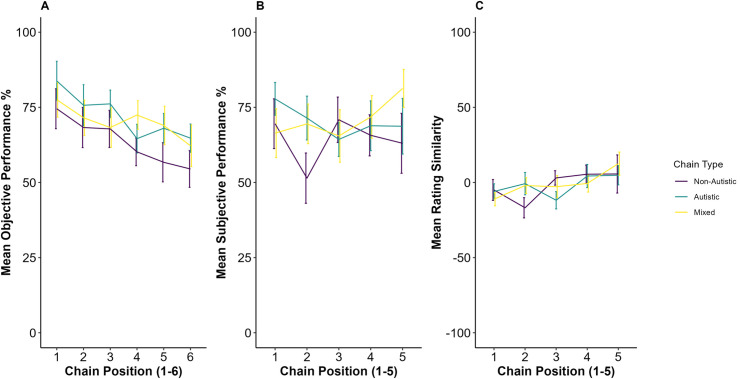
Panel A: Mean objective performance (±1 SE) achieved for six successive participants and chain type (non-autistic, autistic, mixed). Panel B: Mean Subjective Performance (±1 SE) rating given for five successive participants and Chain Type (Non-Autistic, Autistic, Mixed). Panel C: Mean Rating Similarity (±1 SE) for five successive participants and Chain Type (Non-Autistic, Autistic, Mixed).

There was no significant difference in Objective Performance between the three chain types: (Fig 3: between Autistic and Non-Autistic chains β = 8.68, SE = 4.52, t(51.3)=1.92, p = 0.06; between Mixed and Non-Autistic chains β = 6.74, SE = 4.51, t(51.0)=1.49, p = 0.14); thus, H1 was not supported. There was a significant effect of chain position on Objective Performance (β = −3.36, SE = 0.94, t(51.6)=−3.56, p < 0.001), indicating that information transmission decreased down the chains as expected. Additionally, there was no significant effect of whether a participant was informed or uninformed about the diagnostic status of their partner on Objective Performance (our exploratory hypothesis; β = 6.49, SE = 3.91, t(51.0)= 1.66, p = 0.10). The full model output, and an additional model including IQ, Age, Ethnicity and Gender (which does not change the findings relating to chain type and chain position), is illustrated in Supplementary S1 and S7 Tables.

### Subjective performance (self-assessment)

To examine whether Subjective Performance was significantly lower in autistic chains, and higher in non-autistic, compared to mixed chains (H2), a standard linear regression was used. The dependent variable was Subjective Performance, and the best fitting model in terms of AIC and VIF included fixed effects of Chain Type, Chain Position (1–5), and Diagnostic Informing. A random intercept for chain ID could not be estimated and was removed. This model predicted task performance with an adjusted (marginal) R² = 0.009 (R-package MuMIn [53]).

There was no significant difference in Subjective Performance between the three groups (between Autistic and Non-Autistic chains β = 6.17, SE = 4.82, t = 1.28, p = 0.20; between Mixed and Non-Autistic chains β = 6.22, SE = 4.79, t = 1,30, p = 0.20); thus, H2 was not supported (Fig 3). There was no effect of Chain Position (β = 0.35, SE = 1.43, t = 0.25, p = 0.81) or whether a participant was informed or uninformed (β = 0.06, SE = 4.16, t = 0.02, p = 0.99). The full model output, and an additional model including IQ, Age, Ethnicity and Gender (which does not change the findings relating to chain type and chain position), is illustrated in Supplementary S2 and S8 Tables.

### Rating similarity (similarity of self-assessment)

To examine whether Rating Similarity was lower in autistic chains compared to non-autistic and mixed chains (H3), linear mixed effects models were used. The best fitting model by AIC and VIF for the dependent variable of rating similarity included fixed effects for Chain Type, Chain Position (1–5), and a random intercept for Chain ID. In the above analyses of objective and subjective performance (variables from which Rating Similarity was derived) there was no main effect of Diagnostic Informing (Informed, Uninformed) therefore we did not include this variable as an additional predictor in the current model. This model predicted task performance with an adjusted (marginal) R² = 0.04 and adjusted (conditional) R² = 0.10 (R-package MuMIn(54]).

There was no significant difference in Rating Similarity between the autistic, non-autistic and mixed chains (between Autistic and Non-Autistic chains β = −0.31, SE = 4.74, t(51.7)=−0.07, p = 0.95; between Mixed and Non-Autistic chains β = 0.39, SE = 4.72, t=(50.9)=0.08, p = 0.93); thus, H3 was not supported (Fig 3). There was no effect of Chain Position (β = 3.93, SE = 1.22, t(207.5)=3.22, p < 0.01). The full model output is in S3 Table.

We also performed post-hoc analyses considering individual diagnostic status (autistic or non-autistic), rather than diffusion chain group, and the effect of social context (i.e., whether an individual interacted with someone of the same or different neurotype as themselves) on objective performance, subjective performance, and rating similarity. These analyses were in line with the findings presented above and can be viewed in S4–S6 Tables.

## Discussion

Previous research using a verbal task and diffusion chain paradigm found that autistic people can transmit information as effectively as non-autistic people [11]. However, when autistic and non-autistic people were part of a mixed chain, information sharing degraded and less information was transmitted. This challenges the characterisation of autistic individuals as universally deficient in social communication [1,54,55]. However, it is important to examine whether a similar pattern of results will arise using a different type of task therefore we tested whether these findings would generalise when primarily visuospatial, rather than verbal, information is transmitted between participants. Additionally, we examined whether being aware of the diagnostic status of a participant affected task performance, and whether there were differences in how autistic and non-autistic people judged their own performance in the context of same and different neurotype information transfer.

As hypothesised, there was no difference in the amount of information transmitted by autistic and non-autistic chains – autistic and non-autistic people did not differ in their ability to share information with others of the same neurotype. This finding supports the results of Crompton et al. [11] in a larger, multi-site sample, and indicates that the previous result was not an artifact of the specific (language-based) content of the task used but is generalisable to the transmission of visuospatial information. Furthermore, our results challenge the core deficit theory of autism [56], social communication and interaction deficits cited in the diagnostic criteria (DSM-5 [55]), and social cognitive characterisations of autistic individuals as uniformly deficient in social communication [54]. We instead demonstrate that autistic people have effective communication skills and can share information of different types amongst one another in dyads.

However, we found no evidence to support the hypothesis that mixed chains transmit less information than single neurotype chains. As outlined previously, it is possible that the language-based task used in Crompton et al. [11] resulted in the communication breakdown observed in these chains. Though in the current study, participants communicated verbally whilst completing the task – for example, to support their visual demonstrations with verbal descriptions of how to perform certain actions, or to provide encouragement – the task that participants performance was measured by was visuospatial. It is therefore feasible that reducing the requirement for language use – both in terms of the information to be transmitted and relaying this information to one’s partner – may have increased communication compatibility in the mixed chains, increasing resilience to mismatches in language use and communication style. Moreover, the visuospatial nature of the task may have reduced any disadvantage autistic participants (who often have strong visuospatial abilities [57,58]) experienced when being required to verbalise information to transmit it.

We also found no evidence that diagnostic disclosure affected the amount of information transmitted and self-assessment of performance. However, we cannot rule out the possibility that this was due to the specific nature of our task, e.g., that it was relatively structured, goal orientated, and involved interacting with an object, in contrast to previous studies which have focussed on social behaviour and conversation [8,9]. Moreover, participants in our study were aware that we were investigating autistic and non-autistic communication and thus potentially more accepting of differences in social interaction regardless of whether they were informed of their partner’s diagnostic status. Future research should continue to investigate the effect of diagnostic informing in different communicative contexts and less structured, more creative tasks.

The current study had a larger and more heterogeneous sample than Crompton et al. [11]: participants were recruited from three, multi-national sites using a range of recruitment avenues and our sample included both clinically diagnosed and self-identifying autistic participants with age of diagnosis and IQ spanning a wide range. Though our analysis examined the effect of neurotype matching/mismatching on information transmission, matches/mismatches in individual characteristics such as age and gender may also affect social interaction and communication [59,60]. The increased heterogeneity in our sample may thus have introduced noise, overshadowing the effect of neurotype differences and leading to results which differed from those of Crompton et al. Our results revealed some evidence of this: in our models for objective performance and rating similarity, the random effects (i.e., individual makeup of specific diffusion chains) explained more of the variance in objective performance than the fixed effects of chain type and chain position. This shows that the match/mismatch of these factors, in addition to an individual’s autism status, may have a strong influence on social interaction and information transmission. Future work should focus on examining the effects of characteristics such as age, gender, IQ and ethnicity on information transmission in autistic and non-autistic groups.

We attempted to match our autistic and non-autistic participants on age, gender, ethnicity and IQ but as groups significantly differed in these factors (see Methods) we included them as control variables in post-hoc analyses of objective and subjective performance. The results from models including and excluding such variables did not differ (see Supplementary).

We did not find support for our hypotheses related to participants’ ratings of their own performance, or the similarity of these perceptions to their objective performance. Participants in all chain types rated their performance to be of a similar standard (H2) and did not differ in how accurate these ratings were in comparison to objective performance (H3). We thus demonstrated that, in the context presented, autistic people did not show a reduced ability for self-assessment. The literature on self-assessment and autism is in its infancy, but a small body of work shows that autistic people are less accurate than non-autistic people at assessing their own mental state and thus show poorer metacognitive performance [45,61–64]. Our finding of a lack of differentiation between autistic and non-autistic self-assessment similarity contrasts with these findings, however this is an indirect comparison as in our study participants self-assessed *what* they did [e.g., *I did what I was meant to do*] rather than their own mental state or cognitive abilities, although the two are undoubtedly related.

Autistic people have been shown to be less accurate than non-autistic people when self-assessing performance on social, but not general, cognitive tasks [38]. The task used in our study likely drew on more general cognitive abilities (e.g., working memory [65–67]; visuospatial abilities [68,69]) in terms of the requirement to manipulate prism-shaped wedges to create a dog shape. However, it also took place in a social context, with one participant demonstrating production of a dog shape to another, therefore social cognitive skills (e.g., ability to understand partner perspective [70]) were likely also of value. Considering our task required this mix of cognitive skills, we cannot confirm whether our findings on self-assessment align with those of DeBrabander et al. [38]. Additionally, DeBrabander et al. [38] used a direct measure to assess both objective and subjective performance (i.e., participants were asked how many items they thought they got correct), and this was compared to their actual performance when assessing the similarity of self-assessment. However, we used a post-hoc definition of subjective performance, instead asking participants to rate more general statements about their performance (see previous paragraph) which may have confounded our objective and subjective comparison. Future studies could instead ask participants to provide a percentage rating of how closely the dog they produce matches that of the model, improving the alignment of objective and subjective performance.

Our task was interactive and allowed for unrestricted conversation between dyads therefore participants may have varied in the amount of verbal and visual instruction they provided (e.g., describing actions, physically demonstrating a manipulation). Furthermore, the partner of a participant creating a dog shape (i.e., Participant 2 if Participant 1 was creating) was able to provide comments/feedback on their partner’s performance. As feedback was given whilst the task was being completed, it occurred before the participant creating the dog shape was asked to self-rate performance and may have influenced their rating. For example, if positive feedback on poor performance was utilised it could have led to an over-inflated performance rating and thus reduced self-assessment similarity. Analysing the content of the comments/feedback provided is beyond the scope of this article, but future work could look at this more closely to discern whether it had a positive or negative bias and the effect of these biases on self-assessment.

A further potential explanation for the absence of a difference in self-assessment similarity in autistic and non-autistic participants is the visuospatial nature of the task, which may have drawn on autistic strengths [57,58]; autistic people might have also been more likely to be familiar with this type of task. These factors could have reduced anxiety, and increased confidence, in autistic participants, resulting in higher and more accurate self-ratings. Relatedly, the fact that autistic participants had a physical object to interact with may have had additional anxiety-reducing effects and decreased social pressure [71,72], boosting task confidence and resulting in self-assessment similarity which was greater than previous studies suggest. To help us understand the influence of task context on self-assessment similarity, future work could compare self-assessment in an information transmission task with substantial language demands to that found in the current study.

The task used in the current study demonstrated a diffusion effect, indicating that transfer of visuospatial, like verbal, information [11] is subject to degradation over the course of a chain among autistic and non-autistic people. However, the narrative and visuospatial tasks are not perfectly analogous for several important reasons. In the current study we measured task objective performance relative to the original stimulus (i.e., the dog shape shown to Participant 1), paralleling Crompton et al. [11]. This enabled us to track loss of information along the diffusion chain. However, it was possible for an individual participant (2–6) to improve on the performance of the previous participant (e.g., happening upon the solution initially shown in the video through trial and error), therefore our task is likely to have involved both information loss and generation in relation to the task goal. This was more likely than in a narrative task in which the end goal is to reproduce the story told by the previous participant. It is therefore possible that in the current task measuring performance relative to the original stimulus failed to capture some of the subtly in participant behaviour – e.g., whether there were interesting neurotype-specific differences in the extent to which participants tried to re-create and/or improve upon the dog shape created by their partner, or perhaps discounted this and attempted to create their own from scratch. Future work could address this by using a task in which there is a lower likelihood of participants 2–6 independently generating the solution shown to Participant 1, or by using a more open-ended visuospatial task in which the first participant is not shown a pre-determined solution.

## Conclusion

This study confirmed that previous findings [11] on autistic and non-autistic information transmission generalised to a different type of task. These findings challenge stereotypes about autistic social interaction [1,54], revealing that autistic and non-autistic people in same-neurotype interactions can transmit certain types of verbal and non-verbal information as successfully as one another, and thus that autistic social interaction difficulties may be context specific.

These findings lend further support to the idea that autistic communication (in this case, communication with lower verbal demands) can be as successful as non-autistic interaction. Thus, it is more useful to consider autistic communication as being of a different style, rather than deficient in comparison to, non-autistic communication.

However, this study did not find the expected breakdown in information transmission in mixed-neurotype interactions. Future research may examine whether this difference in findings was due to the type of task used in the current study. It may be that tasks that are less verbally demanding reduce the differences in communication style between autistic and non-autistic people, or perhaps reduce the social anxiety, or masking behaviours that may be heightened in mixed pairs and chains. This could have been the cause of mixed groups performing similarly to non-autistic and autistic groups. However, further work is needed to examine if this is the case, and if so, whether this finding can be translated to the real world, to enhance real-world collaborative problem solving and learning environments, or to design more inclusive and effective practices.

Additionally, in contrast to past work on metacognition [44,61–64] and self-assessment [38], we did not find autistic people to show reduced self-assessment ability. However, measurement and methodological differences, as well as potential differences in task cognitive requirements, made it difficult to make direct comparisons and this should be addressed in future work.

## Supporting information

S1 Fig Photographs of dog shapes produced by three of the participants. The prism-shaped wedges in the correct position are denoted by the numbers superimposed on top of the images. Participants in images A, B and C scored 20/24, 9/24 and 14/24 respectively.
(TIF)

S1 Table Output of the objective performance regression model. (DOCX)

S2 Table Output of the subjective performance regression model. (DOCX)

S3 Table Output of the rating similarity regression model. (DOCX)

S4 Table Output of the exploratory analysis objective performance regression model. (DOCX)

S5 Table Output of the exploratory analysis subjective performance regression model. (DOCX)

S6 Table Output of the exploratory analysis rating similarity regression model. (DOCX)

S7 Table Output of the post hoc objective performance regression model. (DOCX)

S8 Table Output of the post hoc subjective performance regression model. (DOCX)

S1 File Inclusivity-in-global-research-questionnaire. (DOCX)
