# Supplementary figures and images for: Visuospatial information transfer and task self-assessment within and between autistic and non-autistic adults
Source: PLoS One. 2025 Aug 14;20(8):e0329825. doi: 10.1371/journal.pone.0329825 (PMC12352780; doi:10.1371/journal.pone.0329825)

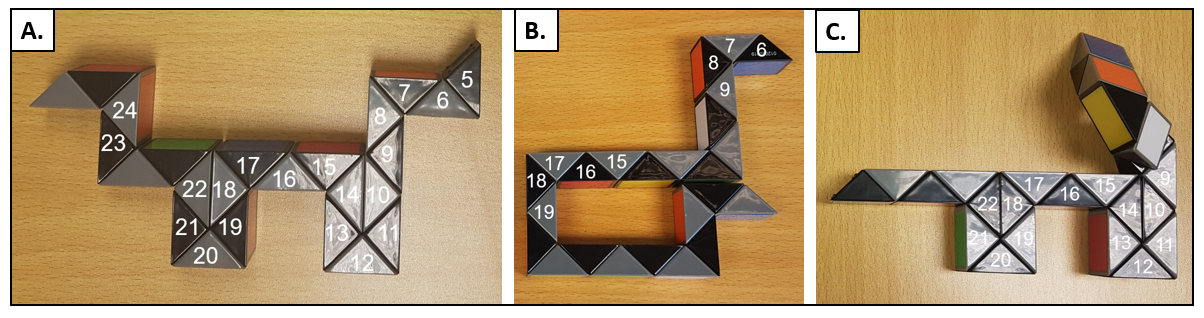

Supplement: S1 Fig — The prism-shaped wedges in the correct position are denoted by the numbers superimposed on top of the images. Participants in images A, B and C scored 20/24, 9/24 and 14/24 respectively. (TIF) [file pone.0329825.s001.tif]
