# Supplementary material for: Visuospatial information transfer and task self-assessment within and between autistic and non-autistic adults
Source: PLoS One. 2025 Aug 14;20(8):e0329825. doi: 10.1371/journal.pone.0329825 (PMC12352780; doi:10.1371/journal.pone.0329825)
Supplement: S1 Table — (DOCX) [file pone.0329825.s002.docx]

**Objective Performance**

|  | Estimate (β) | Std. Error | df | t value | P value |
| --- | --- | --- | --- | --- | --- |
| Intercept (Chain Type = Non-Autistic; Diagnostic Informing = Informed) | 73.123 | 4.715 | 79.162 | 15.509 | <0.001^*^ |
| Chain Type = Autistic | 8.676 | 4.519 | 51.259 | 1.920 | 0.061 |
| Chain Type = Mixed | 6.743 | 4.514 | 51.040 | 1.494 | 0.141 |
| Chain Position | -3.360 | 0.945 | 51.570 | -3.556 | <0.001^*^ |
| Diagnostic Informing = Uninformed | 6.495 | 3.907 | 50.966 | 1.662 | 0.103 |

**Table S1.** Output of the *Objective Performance* regression model.
