# Supplementary material for: Visuospatial information transfer and task self-assessment within and between autistic and non-autistic adults
Source: PLoS One. 2025 Aug 14;20(8):e0329825. doi: 10.1371/journal.pone.0329825 (PMC12352780; doi:10.1371/journal.pone.0329825)
Supplement: S2 Table — (DOCX) [file pone.0329825.s003.docx]

**Subjective Performance**

|  | Estimate (β) | Std. Error | t value | P value |
| --- | --- | --- | --- | --- |
| Intercept (Chain Type = Non-Autistic; Diagnostic Informing = Informed) | 63.142 | 5.537 | 11.404 | <0.001^*^ |
| Chain Type = Autistic | 6.172 | 4.818 | 1.281 | 0.201 |
| Chain Type = Mixed | 6.223 | 4.791 | 1.299 | 0.195 |
| Chain Position | 0.354 | 1.435 | 0.247 | 0.805 |
| Diagnostic Informing = Uninformed | 0.061 | 4.161 | 0.015 | 0.988 |

**Table S2.** Output of the *Subjective Performance* regression model.
