# Supplementary material for: Visuospatial information transfer and task self-assessment within and between autistic and non-autistic adults
Source: PLoS One. 2025 Aug 14;20(8):e0329825. doi: 10.1371/journal.pone.0329825 (PMC12352780; doi:10.1371/journal.pone.0329825)
Supplement: S3 Table — (DOCX) [file pone.0329825.s004.docx]

**Rating Similarity**

|  | Estimate (β) | Std. Error | df | t value | P value |
| --- | --- | --- | --- | --- | --- |
| Intercept (Chain Type = Non-Autistic) | -13.270 | 4.849 | 170.814 | -2.737 | 0.007^*^ |
| Chain Type = Autistic | -0.311 | 4.740 | 51.723 | -0.066 | 0.948 |
| Chain Type = Mixed | 0.392 | 4.719 | 50.880 | 0.083 | 0.934 |
| Chain Position | 3.932 | 1.220 | 207.496 | 3.223 | 0.001^*^ |

**Table S3.** Output of the *Rating Similarity* regression model.
