# Supplementary material for: Visuospatial information transfer and task self-assessment within and between autistic and non-autistic adults
Source: PLoS One. 2025 Aug 14;20(8):e0329825. doi: 10.1371/journal.pone.0329825 (PMC12352780; doi:10.1371/journal.pone.0329825)
Supplement: S4 Table — (DOCX) [file pone.0329825.s005.docx]

**Exploratory Analyses Regression Models**

**Objective Performance**

|  | Estimate (β) | Std. Error | df | t value | P value |
| --- | --- | --- | --- | --- | --- |
| Intercept (Autism Status = Non-Autistic; Social Context = Different) | 80.229 | 4.836 | 88.636 | 16.591 | <0.001^*^ |
| Autism Status = Autistic | 3.497 | 3.081 | 191.119 | 1.135 | 0.258 |
| Social Context = Same | -2.393 | 3.996 | 51.375 | -0.599 | 0.552 |
| Chain Position | -3.341 | 0.944 | 51.617 | -3.540 | <0.001^*^ |

**Table S4.** Output of the Exploratory Analysis *Objective Performance* regression model.
