# Supplementary material for: Visuospatial information transfer and task self-assessment within and between autistic and non-autistic adults
Source: PLoS One. 2025 Aug 14;20(8):e0329825. doi: 10.1371/journal.pone.0329825 (PMC12352780; doi:10.1371/journal.pone.0329825)
Supplement: S5 Table — (DOCX) [file pone.0329825.s006.docx]

**Exploratory Analyses Regression Models**

**Subjective Performance**

|  | Estimate (β) | Std. Error | t value | P value |
| --- | --- | --- | --- | --- |
| Intercept (Autism Status = Non-Autistic; Social Context = Different) | 68.232 | 5.725 | 11.919 | <0.001^*^ |
| Autism Status = Autistic | 2.359 | 3.925 | 0.601 | 0.548 |
| Social Context = Same | -3.120 | 4.151 | -0.752 | 0.453 |
| Chain Position | 0.335 | 1.436 | 0.233 | 0.816 |

**Table S5.** Output of the Exploratory Analysis *Subjective Performance* regression model.
