# Supplementary material for: Visuospatial information transfer and task self-assessment within and between autistic and non-autistic adults
Source: PLoS One. 2025 Aug 14;20(8):e0329825. doi: 10.1371/journal.pone.0329825 (PMC12352780; doi:10.1371/journal.pone.0329825)
Supplement: S6 Table — (DOCX) [file pone.0329825.s007.docx]

**Exploratory Analyses Regression Models**

**Rating Similarity**

|  | Estimate (β) | Std. Error | df | t value | P value |
| --- | --- | --- | --- | --- | --- |
| Intercept (Autism Status = Non-Autistic; Social Context = Different) | -11.896 | 5.219 | 87.380 | -2.279 | 0.025^*^ |
| Autism Status = Autistic | -2.823 | 3.423 | 130.126 | -0.825 | 0.411 |
| Social Context = Same | 0.047 | 3.907 | 51.352 | 0.012 | 0.990 |
| Chain Position | 3.949 | 1.516 | 50.904 | 2.605 | 0.012^*^ |

**Table S6.** Output of the Exploratory Analysis *Rating Similarity* regression model.
