# Supplementary material for: Visuospatial information transfer and task self-assessment within and between autistic and non-autistic adults
Source: PLoS One. 2025 Aug 14;20(8):e0329825. doi: 10.1371/journal.pone.0329825 (PMC12352780; doi:10.1371/journal.pone.0329825)
Supplement: S7 Table — (DOCX) [file pone.0329825.s008.docx]

|  | Estimate (β) | Std. Error | df | t value | P value |
| --- | --- | --- | --- | --- | --- |
| Intercept (Chain Type = Non-Autistic; Diagnostic Informing = Informed, Ethnic Group = White, Gender = Man) | 73.357 | 6.149 | 114.932 | 11.931 | <0.001^*^ |
| Chain Type = Autistic | 6.347 | 5.025 | 63.425 | 1.263 | 0.211 |
| Chain Type = Mixed | 4.612 | 4.781 | 53.614 | 0.965 | 0.339 |
| Chain Position | -3.082 | 1.287 | 65.240 | -2.395 | 0.020^*^ |
| Diagnostic Informing = Uninformed | 5.716 | 4.081 | 50.777 | 1.401 | 0.167 |
| IQ | 5.491 | 1.558 | 217.754 | 3.526 | <0.001^*^ |
| Age (years) | -1.741 | 1.809 | 158.278 | -0.962 | 0.337 |
| Ethnic Group = Asian | -1.9773 | 3.982 | 225.138 | -0.497 | 0.620 |
| Ethnic Group = Black | -0.640 | 8.222 | 200.955 | -0.078 | 0.938 |
| Ethnic Group = Hispanic | 9.926 | 11.014 | 194.471 | 0.901 | 0.369 |
| Ethnic Group = Mixed/Multiple Ethnicities | 3.014 | 6.324 | 230.166 | 0.477 | 0.634 |
| Ethnic Group = Other | -23.082 | 10.581 | 200.821 | -2.181 | 0.030^*^ |
| Gender = Non-Binary/Gender Neutral | -3.388 | 5.771 | 226.819 | -0.587 | 0.558 |
| Gender = Prefer Not to Disclose | -1.341 | 12.707 | 223.955 | -0.105 | 0.916 |
| Gender = Prefer to Self-Describe | -3.265 | 17.426 | 226.423 | -0.187 | 0.852 |
| Gender = Woman | 2.167 | 4.435 | 210.352 | 0.489 | 0.626 |

**Table S7.** Output of the post hoc *Objective Performance* regression model.
