# Supplementary material for: Visuospatial information transfer and task self-assessment within and between autistic and non-autistic adults
Source: PLoS One. 2025 Aug 14;20(8):e0329825. doi: 10.1371/journal.pone.0329825 (PMC12352780; doi:10.1371/journal.pone.0329825)
Supplement: S8 Table — (DOCX) [file pone.0329825.s009.docx]

|  | Estimate (β) | Std. Error | t value | P value |
| --- | --- | --- | --- | --- |
| Intercept (Chain Type = Non-Autistic; Diagnostic Informing = Informed, Ethnic Group = White, Gender = Man) | 59.576 | 7.195 | 8.281 | <0.001^*^ |
| Chain Type = Autistic | 2.894 | 5.486 | 0.528 | 0.598 |
| Chain Type = Mixed | 1.681 | 5.068 | 0.332 | 0.740 |
| Chain Position | 1.609 | 1.576 | 1.021 | 0.308 |
| Diagnostic Informing = Uninformed | -0.865 | 4.291 | -0.201 | 0.840 |
| IQ | 6.273 | 2.081 | 3.014 | 0.003^*^ |
| Age (years) | -1.931 | 2.200 | -0.878 | 0.381 |
| Ethnic Group = Asian | -1.395 | 5.268 | -0.265 | 0.791 |
| Ethnic Group = Black | 1.145 | 10.963 | 0.104 | 0.917 |
| Ethnic Group = Hispanic | 6.068 | 14.610 | 0.415 | 0.678 |
| Ethnic Group = Mixed/Multiple Ethnicities | 12.123 | 8.321 | 1.457 | 0.146 |
| Ethnic Group = Other | 7.642 | 14.390 | 0.531 | 0.596 |
| Gender = Non-Binary/Gender Neutral | 4.104 | 7.412 | 0.554 | 0.580 |
| Gender = Prefer Not to Disclose | 6.477 | 16.792 | 0.386 | 0.700 |
| Gender = Prefer to Self-Describe | 13.503 | 22.903 | 0.590 | 0.556 |
| Gender = Woman | 1.540 | 5.550 | 0.277 | 0.782 |

**Table S8.** Output of the post hoc *Subjective Performance* regression model.
