# Supplementary material for: Visuospatial information transfer and task self-assessment within and between autistic and non-autistic adults
Source: PLoS One. 2025 Aug 14;20(8):e0329825. doi: 10.1371/journal.pone.0329825 (PMC12352780; doi:10.1371/journal.pone.0329825)
Supplement: S1 File — (DOCX) [file pone.0329825.s010.docx]

**Visuospatial Information Transfer and Task Self-Assessment Within and Between Autistic and Non-Autistic Adults**

Supplementary Information

**Contents**

[Rubik’s Twist Task Protocol and Participant Instructions 3](#_Toc204627539)

[Coding Scheme: Rubik’s Twist Task Objective Performance 10](#_Toc204627540)

[Output Tables: Main Manuscript Regression Models 12](#_Toc204627541)

[Objective Performance 12](#_Toc204627542)

[Subjective Performance 13](#_Toc204627543)

[Rating Similarity 14](#_Toc204627544)

[Exploratory Analyses Information 14](#_Toc204627545)

[Output Tables: Exploratory Analyses Regression Models 16](#_Toc204627546)

[Objective Performance 16](#_Toc204627547)

[Subjective Performance 16](#_Toc204627548)

[Rating Similarity 17](#_Toc204627549)

[Post Hoc Models Including Additional Predictor Variables for Objective Performance and Subjective Performance 17](#_Toc204627550)

[Objective Performance 18](#_Toc204627551)

[Subjective Performance 20](#_Toc204627552)

# Rubik’s Twist Task Protocol and Participant Instructions

A straight Rubik’s twist is on the table out of reach of the participant, along with an iPad/video which has the Rubik’s twist video stimuli on it.

- - - - The camera is started.

- Participant 1 is brought into the room and asked to sit in the chair on the right side of the table.

- Participant 2 waits outside the room with a VRA.

- Participant 1 is read the Task Instructions as shown in the researcher packs.

These are:

*“I am going to show you a short video. It is going to show you how to make a dog out of this Rubik's twist. You will only get to see this video once.*

*When it’s finished, you’ll have a chance to practice making a dog for around a minute.*

*I’ll then ask (P2)________________________to come into the room, and you will have 3 minutes to show them how to create a dog. Then it’ll be their turn to show the next person.”*

-The researcher then places the iPad/laptop in front of the participant and presses play. The researcher remains in the room while the video plays but must not distract the participant or interact with them in any way while the video is playing. The participant is only allowed to watch this video once.

- When the video has finished, the researcher should pick up the ipad/laptop, pass the Rubik’s twist to the participant and leave the room for 1 minute

- The researcher should then re-enter the room and take the Rubik’s twist from Participant 1 and take a photo of it using the appropriate sheet from the researcher pack (practice sheet).

- The Rubik’s twist should then be straightened/replaced by a straight twist

- The researcher should meet participant 2 outside the room, and show them into the room and ask them to sit in the left-hand seat. Participant 1 remains in the right-hand seat.

- Participant 2 is then read the instructions from the researcher packs. These are:

*(P1)__________________________________ is going to make a dog from this Rubik’s twist.*

*I will come back in 3 minutes but, (P1)__________________________, you can come and get me if you finish before that. (P2)___________________ you’ll then have a chance to practice making a dog for around a minute.*

*I’ll then ask (P3)__________________________to come into the room, and you should show them how to create a dog. Then it’ll be their turn to show the next person.*

- The researcher then sets the timer and leaves to wait outside the room for 3 minutes, while participants 1 and 2 complete the task. The door should be closed during this time to minimise disruptions and distractions.

- When they are finished, Participant 1 should leave the room, as per their instructions, and find the researcher waiting outside OR after 3 minutes the researcher goes into the interaction room, takes the twist to photo and asks P1 to leave.

- The researcher gives P2 a straight twist and leaves the room for a minute whilst P2 practices with the twist.

- During this time, a VRA should have collected Participant 3, and they should be waiting outside the room, ready to go in.

- A VRA should show Participant 1 back to their Participant room and ask them to complete the rapport measures (see below)

- Either a VRA or researcher needs to photo P1s twist ‘dog’ with the relevant sheet of paper from researcher pack and straighten the twist or have a straight twist ready.

- The researcher should re-enter the room after 1 minute and ask Participant 2 to move to the right-hand seat, and then Participant 3 should be shown into the room and asked to sit in the left-hand seat.

- The researcher then reads Participant 3 the instructions from researcher packs. These are:

*(P2)______________________________ is going to make a dog from this Rubik’s twist.*

*I will come back in 3 minutes but, (P2)__________________________, you can come and get me if you finish before that. (P3)_____________________ you’ll then have a chance to practice making a dog for around a minute.*

*I’ll then ask (P4)_________________________to come into the room, and you should show them how to create a dog. Then it’ll be their turn to show the next person.*

- The researcher then leaves the room to wait outside the room, while participants 2 and 3 complete the task. The door should be closed during this time to minimise disruptions and distractions. During this time, a VRA should have collected Participant 4, and they should be waiting outside the room, ready to go in.

- When they are finished, Participant 2 should leave the room, as per their instructions, and find the researcher waiting outside.

- The VRA should show Participant 2 back to their Participant room and ask them to complete the rapport measures (see below)

- While this is happening, the researcher should ask Participant 3 to move to the right-hand seat, and then Participant 4 should be shown into the room and asked to sit in the left-hand seat.

- The researcher then reads Participant 4 the instructions from researcher packs. These are:

(P3)______________________________ is going to make a dog from this Rubik’s twist.

I will come back in 3 minutes but, (P3)__________________________, you can come and get me if you finish before that. (P4)_____________________ you’ll then have a chance to practice making a dog for around a minute.

I’ll then ask (P5)_________________________to come into the room, and you should show them how to create a dog. Then it’ll be their turn to show the next person.

- The researcher should very quickly check the camera is still recording before leaving the room.

- The researcher then leaves the room to wait outside the room, while participants 3 and 4 complete the task. The door should be closed during this time to minimise disruptions and distractions. During this time, a VRA should have collected Participant 5, and they should be waiting outside the room, ready to go in.

- When they are finished, Participant 3 should leave the room, as per their instructions, and find the researcher waiting outside.

- The VRA should show Participant 3 back to their Participant room and ask them to complete the rapport measures (see below for full details

- While this is happening, the researcher should ask Participant 4 to move to the right-hand seat, and then Participant 5 should be shown into the room and asked to sit in the left-hand seat.

- The researcher then reads Participant 5 the instructions from research packs. These are:

*(P4)___________________________ is going to make a dog from this Rubik’s twist.*

*I will come back in 3 minutes but, (P4)__________________________, you can come and get me if you finish before that. (P5)_____________________ you’ll then have a chance to practice making a dog for around a minute.*

*I’ll then ask (P6)_________________________to come into the room, and you should show them how to create a dog. Then it’ll be their turn to show the next person.*

- The researcher then leaves the room to wait outside the room, while participants 4 and 5 complete the task. The door should be closed during this time to minimise disruptions and distractions. During this time, a VRA should have collected Participant 6, and they should be waiting outside the room, ready to go in.

- When they are finished, Participant 4 should leave the room, as per their instructions, and find the researcher waiting outside.

- The VRA should show Participant 4 back to their Participant room and ask them to complete the rapport measures (see below for full details)

- While this is happening, the researcher should ask Participant 5 to move to the right-hand seat, and then Participant 6 should be shown into the room and asked to sit in the left-hand seat.

- The researcher then reads Participant 6 the instructions from research packs. These are:

*(P5)__________________________ is going to make a dog from this Rubik’s twist.*

*When they are finished, (P5)________________________ please come and tell me – I’ll be waiting outside the door.*

*(P6)_____________________ you’ll then have a chance to practice making a dog for around a minute. I’ll then check you’re ready, before asking you to make the dog shape while the camera records you. I will come back in 3 minutes but you can come and get me if you finish before that.*

- The researcher should very quickly check the camera is still recording before leaving the room.

- The researcher then leaves the room to wait outside the room, while participants 5 and 6 complete the task. The door should be closed during this time to minimise disruptions and distractions.

- When they are finished, Participant 5 should leave the room, as per their instructions, and find the researcher waiting outside.

- The VRA should show Participant 5 back to their Participant room and ask them to complete the rapport measures (see below for full details)

- Participant 6 should then create a dog shape to the camera. They may require to be reminded of their instruction when participant 5 leaves.

- When participant 6 is finished, they should leave the room as per their instructions. The VRA should show Participant 6 back to their Participant room and ask them to complete the rapport measures (see below for full details)

- The researcher should then go into the Research Room, and stop the recording.

- - NB participants do not have to get the dog shape accurate in this task. They just have to create a dog shape. If participants are getting anxious/stressed about doing it wrong, please reassure them by saying
    - “don’t worry about making it perfect”
    - “you’re just trying to end up with a dog shape”
    - “please don’t let this stress you out – just do the best you can and that’ll be great”

# Coding Scheme: Rubik’s Twist Task Objective Performance

The below scheme was used for scoring the Rubik’s Twist Task objective task performance on a scale of 0-24 (later converted into a percentage score).

You should calculate the number of prism shaped wedges, henceforth “triangles” (as viewed in 2D), in the correct position. There are 24 positions, denoted by numbers 1-24 in (see Manuscript Fig 2). The below points should also be adhered to:

- Individual triangle scored must be in the correct orientation to score a point but the colour is irrelevant e.g., largest side South with point North (piece 12); largest side East with point West (piece 11) etc.
- Individual triangles scored must be in one of the designated areas of the dog's body - ear, head, neck, leg right, body, leg left, and tail (see Excel spreadsheet colour coded sections) e.g., you cannot score 17, 16 and 15 if attached to the bottom of a leg or extending from the head because this then can't be considered a body; 17 and 16 differ from 7 and 6 because, although the triangles are in an identical orientation, they are contained within different body parts.
- The Rubik's Twist can be flipped or rotated into whatever position allows for the highest score but then it should remain fixed throughout scoring. Once rotated all scoring needs to be done in that rotation e.g., you can't score the legs with the twist in one location and then rotate it in order to score the body.
- It is possible (and common) to score some points within a body part but not all e.g., if parts 20, 21 and 22 are in a leg position these points can be scored without requiring parts 19 and 18.
- Gaps or extended sections between body parts do not prevent the next body part along from scoring points i.e., sections do not need to move seamlessly from one to the other. For an example see the two unscored triangles between neck point 9 and leg point 10 in Chain 3, Participant 5.
- "3D" sections (those protruding from the solid surface on which the photo is taken at about a 90-degree angle) should be scored as best as possible - there may be flatter sections within these which it is possible to score. Often these sections will score 0.

**
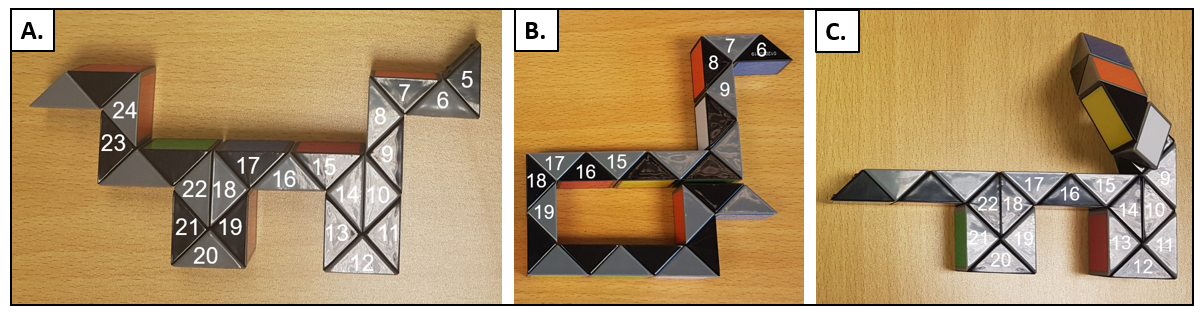
**

Fig S1. Photographs of dog shapes produced by three of the participants. The prism-shaped wedges in the correct position are denoted by the numbers superimposed on top of the images. Participants in images A, B and C scored 20/24, 9/24 and 14/24 respectively.

# Output Tables: Main Manuscript Regression Models

## Objective Performance

|  | Estimate (β) | Std. Error | df | t value | P value |
| --- | --- | --- | --- | --- | --- |
| Intercept (Chain Type = Non-Autistic; Diagnostic Informing = Informed) | 73.123 | 4.715 | 79.162 | 15.509 | <0.001^*^ |
| Chain Type = Autistic | 8.676 | 4.519 | 51.259 | 1.920 | 0.061 |
| Chain Type = Mixed | 6.743 | 4.514 | 51.040 | 1.494 | 0.141 |
| Chain Position | -3.360 | 0.945 | 51.570 | -3.556 | <0.001^*^ |
| Diagnostic Informing = Uninformed | 6.495 | 3.907 | 50.966 | 1.662 | 0.103 |

**Table S1.** Output of the *Objective Performance* regression model.

## Subjective Performance

|  | Estimate (β) | Std. Error | t value | P value |
| --- | --- | --- | --- | --- |
| Intercept (Chain Type = Non-Autistic; Diagnostic Informing = Informed) | 63.142 | 5.537 | 11.404 | <0.001^*^ |
| Chain Type = Autistic | 6.172 | 4.818 | 1.281 | 0.201 |
| Chain Type = Mixed | 6.223 | 4.791 | 1.299 | 0.195 |
| Chain Position | 0.354 | 1.435 | 0.247 | 0.805 |
| Diagnostic Informing = Uninformed | 0.061 | 4.161 | 0.015 | 0.988 |

**Table S2.** Output of the *Subjective Performance* regression model.

## Rating Similarity

|  | Estimate (β) | Std. Error | df | t value | P value |
| --- | --- | --- | --- | --- | --- |
| Intercept (Chain Type = Non-Autistic) | -13.270 | 4.849 | 170.814 | -2.737 | 0.007^*^ |
| Chain Type = Autistic | -0.311 | 4.740 | 51.723 | -0.066 | 0.948 |
| Chain Type = Mixed | 0.392 | 4.719 | 50.880 | 0.083 | 0.934 |
| Chain Position | 3.932 | 1.220 | 207.496 | 3.223 | 0.001^*^ |

**Table S3.** Output of the *Rating Similarity* regression model.

# Exploratory Analyses Information

In the above analyses, the variable Chain Type (Autistic, Non-autistic, Mixed) does not allow us to determine whether any effects present for the Mixed group are driven by the behaviour of autistic participants completing the task with non-autistic partners, or non-autistic participants completing the task with autistic partners (as both were present within the Mixed chain type). We therefore performed post-hoc analyses considering an individuals’ autism status (autistic or non-autistic) and the effect of social context (i.e., whether they are interacting with someone of the same neurotype as themselves or a different neurotype) on Objective Performance, Subjective Performance, and Rating Similarity. In the above analyses, there was a main effect of Chain Position but not Diagnostic Informing, therefore we also included a fixed effect for Chain Position but not Diagnostic Informing in the current analyses.

We performed three separate regression models, one with each of the following dependent variables: Objective Performance, Subjective Performance, and Rating Similarity. For each model, we included the predictor variables of Autism Status (fixed effect; 2 levels; between: Autistic, Non-Autistic), Social Context (fixed effect; 2 levels; between: Same, Different), and Chain Position (fixed effect; within: 1-5). We also included a by-Chain ID random intercept and slope for Chain Position in the Objective Performance and Rating Similarity models - for the Subjective Performance model these could not be estimated and were therefore removed. The Objective Performance and Rating Similarity models predicted task performance with adjusted (marginal) R² values 0.055 and 0.039, and adjusted (conditional) R² values 0.326 and 0.270, respectively; the Subjective Performance model predicted task performance with an adjusted (marginal) R²=-0.008 (R-package MuMIn; Bartoń, 2024).

There was no significant difference in Objective Performance, Subjective Performance, or Rating Similarity according to Autism Status (Autistic or Non-Autistic) or Social Context (Same or Different); see below Tables S4-S6. There was a significant effect of *Chain Position* on Objective Performance (β=-3.34, SE=0.94, t(51.6)= -3.54, p<0.001), indicating that information transmission decreased down the chains as expected (Table S4). There was no effect of *Chain Position* on Subjective Performance (β=0.33, SE=1.44, t=0.23, p=0.82; Table S5), or Rating Similarity (β=3.95, SE=1.52, t(50.9)=2.61, p=0.01; Table S6).

# Output Tables: Exploratory Analyses Regression Models

## Objective Performance

|  | Estimate (β) | Std. Error | df | t value | P value |
| --- | --- | --- | --- | --- | --- |
| Intercept (Autism Status = Non-Autistic; Social Context = Different) | 80.229 | 4.836 | 88.636 | 16.591 | <0.001^*^ |
| Autism Status = Autistic | 3.497 | 3.081 | 191.119 | 1.135 | 0.258 |
| Social Context = Same | -2.393 | 3.996 | 51.375 | -0.599 | 0.552 |
| Chain Position | -3.341 | 0.944 | 51.617 | -3.540 | <0.001^*^ |

**Table S4.** Output of the Exploratory Analysis *Objective Performance* regression model.

## Subjective Performance

|  | Estimate (β) | Std. Error | t value | P value |
| --- | --- | --- | --- | --- |
| Intercept (Autism Status = Non-Autistic; Social Context = Different) | 68.232 | 5.725 | 11.919 | <0.001^*^ |
| Autism Status = Autistic | 2.359 | 3.925 | 0.601 | 0.548 |
| Social Context = Same | -3.120 | 4.151 | -0.752 | 0.453 |
| Chain Position | 0.335 | 1.436 | 0.233 | 0.816 |

**Table S5.** Output of the Exploratory Analysis *Subjective Performance* regression model.

## Rating Similarity

|  | Estimate (β) | Std. Error | df | t value | P value |
| --- | --- | --- | --- | --- | --- |
| Intercept (Autism Status = Non-Autistic; Social Context = Different) | -11.896 | 5.219 | 87.380 | -2.279 | 0.025^*^ |
| Autism Status = Autistic | -2.823 | 3.423 | 130.126 | -0.825 | 0.411 |
| Social Context = Same | 0.047 | 3.907 | 51.352 | 0.012 | 0.990 |
| Chain Position | 3.949 | 1.516 | 50.904 | 2.605 | 0.012^*^ |

**Table S6.** Output of the Exploratory Analysis *Rating Similarity* regression model.

# Post Hoc Models Including Additional Predictor Variables for Objective Performance and Subjective Performance

Linear Mixed Effects Modelling was used to examine the impact of multiple predictor variables on Objective Performance and Subjective Performance, including the covariates of age, gender, IQ, and ethnicity. Here we present analogous models to the ones in the main paper, with the addition of the covariates as follows:

Objective Performance ~ *Chain Type* + *Chain Position* + *Diagnostic Informing* + *IQ* + *Age* + *Ethnic Group* + *Gender* + (1 + *Chain Position* | Chain Number)

Subjective Performance ~ *Chain Type* + *Chain Position* + *Diagnostic Informing* + *IQ* + *Age* + *Ethnic Group* + *Gender*

The aim is to explore how these covariates, alongside core predictors like chain type (autistic, non-autistic, and mixed) and chain position, influence Objective Performance, Subjective Performance, and Rating Similarity.

## Objective Performance

This model predicted task performance with an adjusted (marginal) *R^2^*=0.13 and adjusted (conditional) *R^2^*=0.32 (R-package MuMIn; Bartoń, 2024). As per the model in the main manuscript, there was no significant difference in Objective Performance according to *Chain Type* (between *Chain Type* Autistic and *Chain Type* Non-Autistic (β=6.35, SE=5.03, t(63.4)=1.26, p=0.21); between C*hain Type* Mixed and *Chain Type* Non-Autistic (β=4.61, SE=4.78, t(53.6)=0.97, p=0.34)) and whether a participant was informed or uninformed (Diagnostic Informing; β=5.72, SE=4.08, t(50.8)=1.40, p=0.17), but there was a significant effect of *Chain Position* (β=-3.08, SE=1.29, t(65.2)=-2.40, p=0.02). IQ was also a significant predictor, with higher IQ scores associated with higher task performance (β=5.49, SE=1.56, t(217.8)=3.53, p<0.001). Additionally, ethnic group had a significant effect on task performance (between “Other” ethnic group and “White”: β=-23.08, SE=10.58, t(200.8)=-2.18, p=0.03). The full model output is illustrated in Table S7 below.

|  | Estimate (β) | Std. Error | df | t value | P value |
| --- | --- | --- | --- | --- | --- |
| Intercept (Chain Type = Non-Autistic; Diagnostic Informing = Informed, Ethnic Group = White, Gender = Man) | 73.357 | 6.149 | 114.932 | 11.931 | <0.001^*^ |
| Chain Type = Autistic | 6.347 | 5.025 | 63.425 | 1.263 | 0.211 |
| Chain Type = Mixed | 4.612 | 4.781 | 53.614 | 0.965 | 0.339 |
| Chain Position | -3.082 | 1.287 | 65.240 | -2.395 | 0.020^*^ |
| Diagnostic Informing = Uninformed | 5.716 | 4.081 | 50.777 | 1.401 | 0.167 |
| IQ | 5.491 | 1.558 | 217.754 | 3.526 | <0.001^*^ |
| Age (years) | -1.741 | 1.809 | 158.278 | -0.962 | 0.337 |
| Ethnic Group = Asian | -1.9773 | 3.982 | 225.138 | -0.497 | 0.620 |
| Ethnic Group = Black | -0.640 | 8.222 | 200.955 | -0.078 | 0.938 |
| Ethnic Group = Hispanic | 9.926 | 11.014 | 194.471 | 0.901 | 0.369 |
| Ethnic Group = Mixed/Multiple Ethnicities | 3.014 | 6.324 | 230.166 | 0.477 | 0.634 |
| Ethnic Group = Other | -23.082 | 10.581 | 200.821 | -2.181 | 0.030^*^ |
| Gender = Non-Binary/Gender Neutral | -3.388 | 5.771 | 226.819 | -0.587 | 0.558 |
| Gender = Prefer Not to Disclose | -1.341 | 12.707 | 223.955 | -0.105 | 0.916 |
| Gender = Prefer to Self-Describe | -3.265 | 17.426 | 226.423 | -0.187 | 0.852 |
| Gender = Woman | 2.167 | 4.435 | 210.352 | 0.489 | 0.626 |

**Table S7.** Output of the post hoc *Objective Performance* regression model.

## Subjective Performance

This model predicted task performance with an adjusted (marginal) R²=0.06 (R-package MuMIn; Bartoń, 2024). Note, a random intercept for chain ID could not be estimated and was removed, therefore this model was a standard linear regression with no random effects. As per the model in the main manuscript, there was no significant difference in Subjective Performance between the three groups (between *Chain Type* Autistic and *Chain Type* Non-Autistic (β=2.89, SE=5.49, t=0.53, p=0.60); between C*hain Type* Mixed and *Chain Type* Non-Autistic (β=1.68, SE=5.07, t=0.33, p=0.74)) and no effect of Chain Position (β=1.61, SE=1.58, t=1.02, p=0.31) or whether a participant was informed or uninformed (β=-0.86, SE=4.29, t=-0.20, p=0.84). IQ was a significant predictor, with higher IQ scores associated with higher task performance (β=6.27, SE=2.08, t=3.01, p<0.01). The full model output is illustrated in Table S8 below.

|  | Estimate (β) | Std. Error | t value | P value |
| --- | --- | --- | --- | --- |
| Intercept (Chain Type = Non-Autistic; Diagnostic Informing = Informed, Ethnic Group = White, Gender = Man) | 59.576 | 7.195 | 8.281 | <0.001^*^ |
| Chain Type = Autistic | 2.894 | 5.486 | 0.528 | 0.598 |
| Chain Type = Mixed | 1.681 | 5.068 | 0.332 | 0.740 |
| Chain Position | 1.609 | 1.576 | 1.021 | 0.308 |
| Diagnostic Informing = Uninformed | -0.865 | 4.291 | -0.201 | 0.840 |
| IQ | 6.273 | 2.081 | 3.014 | 0.003^*^ |
| Age (years) | -1.931 | 2.200 | -0.878 | 0.381 |
| Ethnic Group = Asian | -1.395 | 5.268 | -0.265 | 0.791 |
| Ethnic Group = Black | 1.145 | 10.963 | 0.104 | 0.917 |
| Ethnic Group = Hispanic | 6.068 | 14.610 | 0.415 | 0.678 |
| Ethnic Group = Mixed/Multiple Ethnicities | 12.123 | 8.321 | 1.457 | 0.146 |
| Ethnic Group = Other | 7.642 | 14.390 | 0.531 | 0.596 |
| Gender = Non-Binary/Gender Neutral | 4.104 | 7.412 | 0.554 | 0.580 |
| Gender = Prefer Not to Disclose | 6.477 | 16.792 | 0.386 | 0.700 |
| Gender = Prefer to Self-Describe | 13.503 | 22.903 | 0.590 | 0.556 |
| Gender = Woman | 1.540 | 5.550 | 0.277 | 0.782 |

**Table S8.** Output of the post hoc *Subjective Performance* regression model.
